# Special Session Best of 2023: Care of the elderly

**DOI:** 10.1192/j.eurpsy.2024.33

**Published:** 2024-08-27

**Authors:** R. Perneczky

**Affiliations:** Department of Psychiatry and Psychotherapy, LMU Hospital, LMU Munich, Munich, Germany

## Abstract

**Abstract:**

In the field of Alzheimer’s disease disease-modifying therapy, there has been a shift in diagnosis from the later dementia stages towards the earlier stages, with the potential for pre-symptomatic diagnosis. The development of truly ‘disease-modifying’ therapies that target the underlying mechanisms of Alzheimer’s disease has reached late stages of human clinical trials. The primary targets include beta-amyloid, whose presence and accumulation in the brain is thought to contribute to the development of Alzheimer’s disease, and tau protein which, when hyperphosphorylated, results in the self-assembly of tangles of paired helical filaments also believed to be involved in the pathogenesis of Alzheimer’s disease. Therapeutic strategies aimed at preventing Aβ formation, blocking its aggregation into plaques, lowering its soluble levels in the brain, and disassembling existing amyloid plaques are among the main strategies employed to slow the progression of AD. First anti-amyloid antibody treatments have proven effective in late-stage clinical trials and are now being approved for clinical use in some countries, initiating a new are of treatment. In terms of blood-based early diagnosis, the development of in vivo biomarkers has shifted the diagnosis of Alzheimer’s disease from the later dementia stages of disease towards the earlier stages and has introduced the potential for pre-symptomatic diagnosis. Recent study shows promising results for blood tests that could be used to identify Alzheimer’s changes in the brain before the onset of any symptoms, which could result in preventative treatments being used before any memory loss. This presentation will highlight the most exciting development of the past year in the Alzheimer’s disease therapy and diagnosis arena.

**Disclosure of Interest:**

None Declared

